# Effect of Glucocorticoid Receptor Antagonism on Alcohol Self-Administration in Genetically-Selected Marchigian Sardinian Alcohol-Preferring and Non-Preferring Wistar Rats

**DOI:** 10.3390/ijms22084184

**Published:** 2021-04-17

**Authors:** Federica Benvenuti, Nazzareno Cannella, Serena Stopponi, Laura Soverchia, Massimo Ubaldi, Veronica Lunerti, Valentina Vozella, Bryan Cruz, Marisa Roberto, Roberto Ciccocioppo

**Affiliations:** 1 Pharmacology Unit, School of Pharmacy, University of Camerino, 62032 Camerino, Italy; federica.benvenuti@unicam.it (F.B.); nazzareno.cannella@unicam.it (N.C.); serena.stopponi@unicam.it (S.S.); laura.soverchia@unicam.it (L.S.); massimo.ubaldi@unicam.it (M.U.); veronica.lunerti@unicam.it (V.L.); 2 Department of Molecular Medicine, The Scripps Research Institute, La Jolla, CA 92037, USA; vvozella@scripps.edu (V.V.); bcruz@scripps.edu (B.C.); mroberto@scripps.edu (M.R.)

**Keywords:** alcohol use disorder, stress, alcohol preferring rats, glucocorticoids, mifepristone, alcohol self-administration

## Abstract

Alcoholism is a chronically relapsing disorder characterized by high alcohol intake and a negative emotional state during abstinence, which contributes to excessive drinking and susceptibility to relapse. Stress, dysregulation of the hypothalamic-pituitary-adrenal (HPA) axis and alterations in glucocorticoid receptor (GR) function have been linked to transition from recreational consumption to alcohol use disorder (AUD). Here, we investigated the effect of pharmacological antagonisms of GR on alcohol self-administration (SA) using male and female Wistar and Marchigian Sardinian alcohol-preferring (msP) rats, a rodent line genetically selected for excessive alcohol drinking and highly sensitive to stress. Animals were trained to self-administer 10% (*v*/*v*) alcohol. Once a stable alcohol SA baseline was reached, we tested the effect of the GR antagonists mifepristone (0.0, 10, 30 and 60 mg/kg; i.p.) and CORT113176 (0.0, 10, 30 and 60 mg/kg) on alcohol SA. To evaluate whether the effects of the two compounds were specific for alcohol, the two drugs were tested on a similar saccharin SA regimen. Finally, basal blood corticosterone (CORT) levels before and after alcohol SA were determined. Systemic injection with mifepristone dose-dependently reduced alcohol SA in male and female Wistars but not in msPs. Administration of CORT113176 decreased alcohol SA in male and female Wistars as well as in female msPs but not in male msP rats. At the highest dose, mifepristone also reduced saccharin SA in male Wistars and female msPs, suggesting the occurrence of some nonspecific effects at 60 mg/kg of the drug. Similarly, the highest dose of CORT113176 (60 mg/kg) decreased saccharin intake in male Wistars. Analysis of CORT levels revealed that females of both rat lines had higher blood levels of CORT compared to males. Alcohol consumption reduced CORT in females but not in males. Overall, these findings indicate that selective blockade of GR selectively reduces alcohol SA, and genetically selected msP rats are less sensitive to this pharmacological manipulation compared to heterogeneous Wistars. Moreover, results suggest sex differences in response to GR antagonism and the ability of alcohol to regulate GR transmission.

## 1. Introduction

Alcohol use disorder (AUD) is a complex psychiatric condition characterized by excessive drug use, loss of control over its consumption and emergence of a negative emotional state during withdrawal that contribute to relapse [1]. AUD is a major public health problem, and alcohol represents a significant disability and morbidity factor responsible of about three million deaths per year [2].

Stress and dysregulation of related hormones of the hypothalamic-pituitary-adrenal (HPA) axis have been proposed as important factors affecting disease progression [3,4]. The HPA axis represents the primary neuroendocrine network controlling stress response, and its activation in response to external or internal perturbation culminates in the production and release of cortisol in humans and corticosterone (CORT) in rodents [5]. Once released, glucocorticoids act through either the high affinity mineralocorticoid or the low affinity glucocorticoid receptor (GR). GR is highly expressed in several brain regions of the limbic system, in the paraventricular nucleus (PVN) of the hypothalamus and the anterior pituitary gland [6]. Once released, glucocorticoids produce an array of physiological effects to adjust the organism to stressor exposure and are also responsible for termination of their actions via negative feedback inhibition at HPA level [5].

The motivation to drink alcohol is initially driven by positive reinforcement mechanisms, and its consumption is usually linked to recreational purposes. Studies in rodent models mimicking the early stages of alcohol consumption demonstrated that CORT administration increased alcohol self-administration (SA) [7,8,9] whereas adrenalectomy decreased it [10]. Noteworthy is that alcohol drinking was recovered by corticosterone replacement, suggesting that glucocorticoids facilitate alcohol reinforcement [10]. As a result of chronic alcohol drinking, the excessive and protracted activation of the HPA axis may lead to its dysregulation. This contributes to the surge of compulsive alcohol drinking motivated by the need to self-medicate to attenuate the negative symptoms associated with alcohol withdrawal [4,11,12]. Earlier studies demonstrated that alcohol-dependent rats exhibited significant downregulation of GR during acute withdrawal, and GR upregulation during protracted abstinence in several stress/reward related brain areas, suggesting that the GR system may contribute to the progression of AUD [13].

The genetically selected Marchigian Sardinian alcohol-preferring (msP) rat line is a well consolidated animal model to study AUD. In this rat line, anxiety and depressive-like traits have been cosegregated with high alcohol preference during the selection process. Hence, it is possible that their innate propensity to consume high amounts of alcohol is driven by the attempt to self-medicate from an innate negative affect, specifically mimicking the subpopulation of humans with alcoholism that consume alcohol for tension relief purposes [14,15]. Consistent with this view, earlier studies showed that msP rats carry two single nucleotide polymorphisms in the promoter region of the CRF1 receptor (CRF1-R) leading to hyperactivation of the system that is attenuated by voluntary alcohol consumption [16,17,18]. These mutations have been also associated with a decreased threshold for stress-induced alcohol-seeking and conferred to msP rats higher sensitivity to CRF1-R antagonists [16,19,20]. Noteworthy is that these gene polymorphisms are conserved in the human CRF system and have been correlated with the diagnosis of AUD [21,22]. We also reported that male msP rats displayed dysregulated GABA and glutamate signaling [23,24,25]. Recently, it has been found that male msP rats displayed diminished stress-induced GR phosphorylation at the serine site 232 in the PVN and a constitutive increase in phosphorylated GR levels in the central nucleus of the amygdala (CeA) [26]. The elevation of GR phosphorylation was also observed in the CeA of alcohol-dependent rats during acute withdrawal [27]. In postdependent rats, systemic and intra-CeA administration of mifepristone, a nonselective glucocorticoid and progesterone receptor antagonist, reduced alcohol intake and yohimbine-induced reinstatement of alcohol seeking [27,28].

Currently, it is unknown whether the constitutive alteration of GR levels of msP rats might contribute to their excessive alcohol-drinking phenotype. However, considering that this rat line shows features resembling postdependent rats, we thought it important to explore the effect of pharmacological antagonism of GR on alcohol self-administration by comparing the msP rat line with its Wistar counterpart. Moreover, considering that several sex differences have been described in response to stress and to alcohol, and that the HPA axis function is greater in female rats, in the present study we tested males and females separately [15,29,30,31,32,33].

## 2. Results

### 2.1. Experiment 1.1: Effect of Mifepristone on Alcohol Self-Administration in Male and Female msP and Wistar Rats

**Figure 1:**
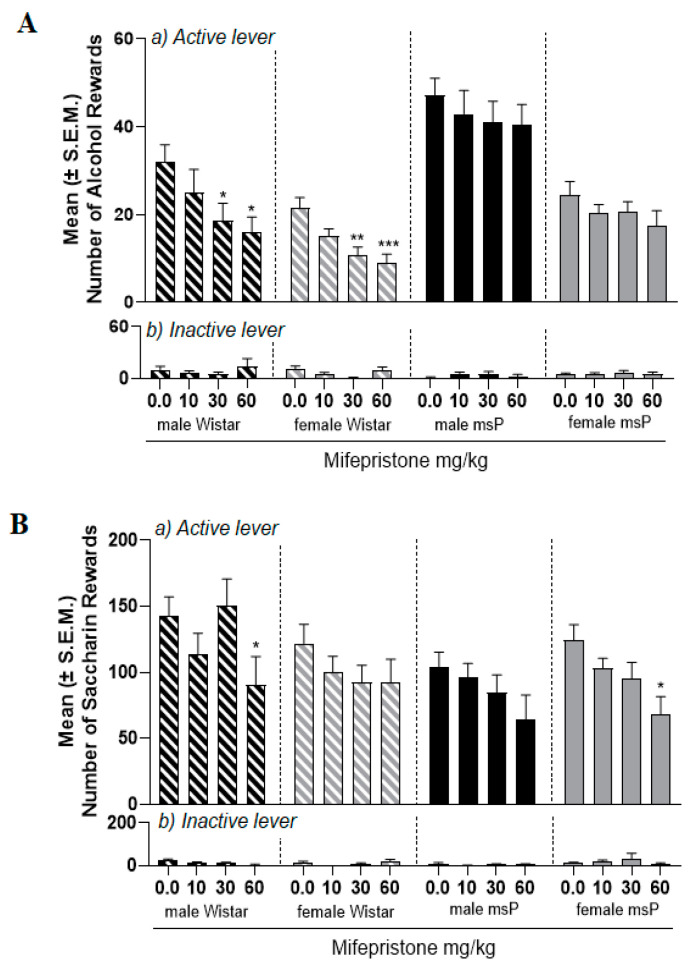
Effect of mifepristone on alcohol and saccharin self-administration in male and female msP and Wistar rats. Male and female msP and Wistar rats were treated with mifepristone (0.0, 10, 30 and 60 mg/kg) i.p., 90 min prior to test session. (**A**) Mifepristone treatment significantly reduced the number of alcohol rewards in male and female Wistars. Drug treatment did not decrease alcohol SA in male and female msPs. (**B**) At the dose of 60 mg/kg, mifepristone significantly reduced saccharin SA in male Wistars and in female msPs. Data are expressed as the mean ± SEM of number of: (**a**) reinforced responses (rewards) at the active lever and (**b**) total responses at the inactive lever. Significant difference from vehicle (0.0 mg/kg): * *p* < 0.05; ** *p* < 0.01; *** *p* < 0.001.

We tested the effect of mifepristone on alcohol SA under Fixed Ratio 1 (FR1) schedule of reinforcement in male and female msP (N = 10/sex) and Wistar (N = 9–10/sex) rats. Experimental subjects received mifepristone (10, 30 and 60 mg/kg) or its vehicle in a counterbalanced within subject Latin square design. A three-way ANOVA revealed an overall effect of treatment [F_(3,35)_ = 7.5; *p* < 0.001], sex [F_(1,35)_ = 55.8; *p* < 0.0001] and strain [F_(1,35)_ = 41.2; *p* < 0.0001]. There was a significant sex x strain interaction [F_(1,35)_ = 10.2; *p* < 0.01], but no other significant interactions. These results reflect higher SA levels in msP, a higher number of rewards by male msP rats and a general reduction of alcohol SA induced by mifepristone. To further evaluate the effect of mifepristone, we carried out single ANOVAs to independently analyze the drug effect on male and female msPs as well as on male and female Wistars. In msP rats no overall effect of treatment in male [F_(3,9)_ = 0.4; *p* > 0.05] or in female rats [F_(3,9)_ = 1.1; *p* > 0.05] was detected. Conversely, an overall significant effect of treatment was detected in male [F_(3,8)_ = 4.0; *p* < 0.05] and female [F_(3,9)_ = 7.5; *p* < 0.01] Wistars. Dunnett’s post hoc analysis showed a significant decrease in the number of alcohol-reinforced responding at doses of 30 mg/kg and 60 mg/kg of mifepristone in both male and female Wistar rats (*p* < 0.05; Figure 1A, upper panel).

A three-way ANOVA applied to inactive lever responding showed no overall effect of treatment [F_(3,35)_ = 0.9; *p* > 0.05], sex [F_(1,35)_ = 0.05; *p* > 0.05] or strain [F_(1,35)_ = 2.3; *p* > 0.05]. Neither interaction was detected (Figure 1A, lower panel).

### 2.2. Experiment 1.2: Effect of Mifepristone on Saccharin Self-Administration in Male and Female msP and Wistar Rats

To control for the selectivity of mifepristone effect on alcohol SA, other groups of male and female msP (N = 6–8/sex) and Wistar (N = 7–8/sex) rats were tested for the effect of mifepristone (10, 30 and 60 mg/kg) or its vehicle on saccharin SA. A three-way ANOVA found a significant effect of treatment [F_(3,25)_ = 8.8; *p* = 0.0001], no effect of sex [F_(1,25)_ = 0.3; *p* > 0.05], no effect of strain [F_(1,25)_ = 0.4; *p* > 0.05] and no interactions. To further explore the effect of mifepristone, data from male and female msPs and male and female Wistars were analyzed separately by single ANOVAs. Results revealed an overall effect of treatment in male Wistars [F_(3,6)_ = 4.7; *p* < 0.05] and female msPs [F _(3,7)_ = 6.4; *p* < 0.01]. Conversely, no overall effect was found in female Wistars [F_(3,7)_ = 1.4; *p* > 0.05] and male msPs [F_(3,5)_ = 1.2; *p* > 0.05]. Dunnet’s post hoc tests showed that 60 mg/kg of mifepristone reduced saccharin SA in both male Wistars and female msPs (*p* < 0.05) (Figure 1B, upper panel).

Analysis of inactive lever responding found no significant overall effect of treatment [F_(3,25)_ = 0.7; *p* > 0.05], sex [F_(1,25)_ = 1.4; *p* > 0.05], strain [F_(1,25)_ = 0.005; *p* > 0.05] and no interactions (Figure 1B, upper panel).

### 2.3. Experiment 2.1: Effect of CORT113176 on Alcohol Self-Administration in Male and Female msP and Wistar Rats

**Figure 2:**
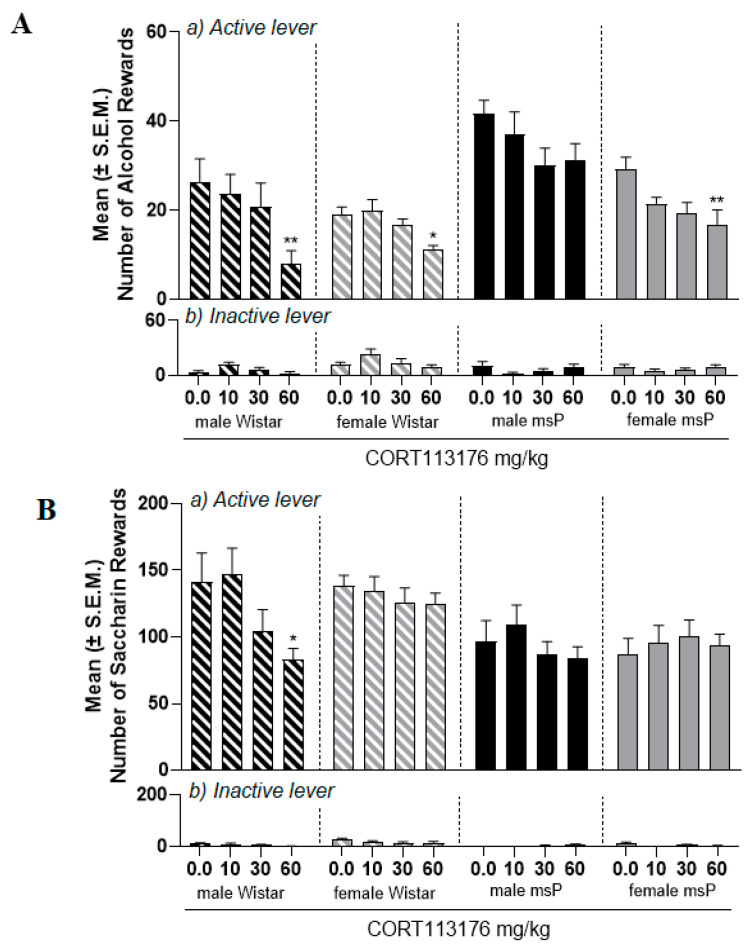
Effect of CORT113176 on alcohol and saccharin self-administration in male and female msP and Wistar rats. Male and female msP and Wistar rats were treated with CORT113176 (0.0, 10, 30 and 60 mg/kg) i.p., 90 min prior to test session. (**A**) CORT113176 treatment significantly reduced the number of alcohol rewards in male and female Wistars and in female msP rats. (**B**) CORT113176 at the dose of 60 mg/kg significantly reduced saccharin SA in male Wistar rats only. Data are expressed as the mean ± SEM of number of: (**a**) reinforced responses at the active and (**b**) total responses at inactive lever. Significant difference from vehicle (0.0 mg/kg): ** *p* < 0.01; * *p* < 0.05.

Mifepristone is a GR antagonist that also has activity on the progesterone receptor. To confirm that effects observed were specifically mediated by GR antagonism, we tested CORT113176, which is another more selective GR antagonist [27]. Once a stable baseline of alcohol SA was reached, male and female msP (N = 9–10/sex) and Wistar (N = 10/sex) rats were treated with CORT113176 (10, 30, 60 mg/kg) or its vehicle. A three-way ANOVA revealed an overall effect of treatment [F_(3,35)_ = 11.1; *p* < 0.0001], sex [F_(1,35)_ = 16.04; *p* < 0.001], strain [F_(1,35)_ = 24.6; *p* < 0.0001] and sex x strain interaction [F_(1,35)_ = 6.3; *p* < 0.05], but no other significant interactions (Figure 2A, upper panel). At this point we conducted single ANOVAs to further determine the effect of CORT113176 on male and female msPs and male and female Wistars. Results showed an overall effect of treatment in male Wistars [F_(3,9)_ = 4.4; *p* < 0.05], female Wistars [F_(3,9)_ = 4.5; *p* < 0.05] and female msPs [F_(3,9)_ = 4.0, *p* < 0.05]. No effect was found in male msP [F_(3,8)_ = 1.9; *p* > 0.05] rats. Dunnet’s post hoc tests revealed that at 60 mg/kg, CORT113176 decreased alcohol SA in male (*p* < 0.01) and female Wistars (*p* < 0.05) as well as female msPs (*p* < 0.01).

Analysis of the inactive lever found no significant overall effect of treatment [F_(3,35)_ = 0.8; *p* > 0.05] and strain [F_(1,35)_ = 3.7; *p* > 0.05] but an overall effect of sex [F_(1,35)_ = 7.7; *p* < 0.01], treatment x strain [F_(1,35)_ = 5.6; *p* < 0.01] and sex x strain interaction [F_(1,35)_ = 5.5; *p* < 0.05] was observed (Figure 2A, lower panel).

### 2.4. Experiment 2.2: Effect of CORT113176 on Saccharin Self-Administration in Male and Female msP and Wistar Rats

We next verified the specificity of action of CORT113176 by testing its effect on saccharin SA in male and female msP (N = 9–10/sex) and Wistar (N = 9–10/sex) rats. Three-way ANOVA demonstrated a significant effect of treatment [F_(3,34)_ = 5.2; *p* < 0.01], strain [F_(1,34)_ = 10.3; *p* < 0.01] and treatment x sex interaction [F_(3,102)_ = 3,4; *p* < 0.05] (Figure 2B, upper panel). When single ANOVAs were carried out, we found an overall effect of CORT113176 on saccharin SA only in male Wistar rats [F_(3,8)_ = 4.5; *p* < 0.05]. No drug effect was detected in female Wistars [F_(3,9)_ = 0.7; *p* > 0.05], male msPs [F_(3,8)_ = 1.7; *p* > 0.05] and in female msPs [F_(3,9)_ = 0.4; *p* > 0.05].

Analysis of the inactive lever found no significant overall effect of treatment [F_(3,34) =_ 2.7; *p* > 0.05], but there was a significant effect of sex [F_(1,34) =_ 9.3; *p* < 0.01], strain [F_(1,34) =_ 15.4; *p* < 0.01] and treatment x strain interaction [F_(3,102) =_ 2.9; *p* < 0.05] (Figure 2B, lower panel).

### 2.5. Experiment 3: Blood CORT Levels Following Alcohol Self-Administration in Male and Female msP and Wistar Rats

**Figure 3:**
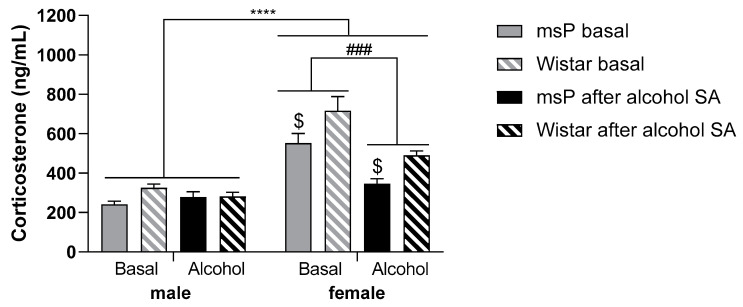
Blood corticosterone (CORT) levels under basal conditions and after alcohol SA sessions in male and female msP and Wistar rats. Females displayed significantly higher blood CORT levels than males independently of rat strain. Female Wistars had higher CORT levels than female msPs. Alcohol consumption decreased basal CORT levels in female animals only. In both rat lines, CORT levels of male rats remained unchanged following alcohol SA. Data are presented as mean ± SEM. Main effect of sex: **** *p* < 0.0001; main effect of sex x alcohol condition: ### *p* < 0.001; $ *p* < 0.05 vs. msP same condition and sex (sex x strain interaction).

Finally, we assessed the blood CORT levels under basal conditions and after alcohol SA in male and female msP (N = 6/sex) and Wistar rats (N = 8–7/sex). Three-way ANOVA revealed a main effect of sex [F_(1,23)_ = 84.5; *p* < 0.0001], alcohol condition [F_(1,23)_ = 19.5; *p* < 0.001], strain [F_(1,23)_ = 13.8; *p* < 0.01], sex x alcohol condition interaction [F_(1,23)_ = 18.3; *p* < 0.001] and sex x strain interaction [F_(1,23)_ = 4.4; *p* < 0.05]. Female rats from both genotypes displayed persistently higher levels of CORT compared to male rats in both conditions. Female Wistar rats showed higher CORT levels than female msPs (*p* < 0.001). Alcohol consumption in a SA session decreased CORT levels only in female animals (*p* < 0.001). In male rats, blood CORT concentrations were not affected by alcohol SA (Figure 3).

## 3. Discussion

The present study investigated the effect of glucocorticoid receptor antagonism on alcohol drinking in genetically-selected msP rats in comparison with nonselected Wistar rats. To summarize, we found that mifepristone administration reduced alcohol SA in both male and female Wistar rats, but not msPs, at similar dose ranges utilized in previous studies measuring alcohol SA in dependent Wistar rats [27]. The ability of mifepristone to reduce alcohol SA was apparent at the intermediate dose of 30 mg/kg, while higher doses (60 mg/kg) appeared to produce nonselective reductions of saccharin SA, suggesting the occurrence of nonspecific effects. Given the nonselectivity of mifepristone in antagonizing progesterone receptors also, we tested the selective CORT113176 compound that targets GR to confirm whether reducing alcohol SA requires specificity for the GR. Consistent with results with mifepristone, CORT113176 significantly reduced alcohol SA in male and female Wistars as well as female msP rats. As for mifepristone, male msPs did not respond to CORT113176 treatment. Furthermore, administration of CORT113176 at the highest dose reduced saccharin SA only in male Wistar rats. Taken together, we suggest that our drug regimen is specific to alcohol SA, since the number of saccharin rewards was not modified in the other groups of rats. However, at high doses, nonspecific inhibition of motivated behavior may emerge. Earlier work demonstrated that mifepristone decreases alcohol consumption in a limited-access two-bottle choice paradigm [34], and intra-CeA infusion of mifepristone reduces alcohol-seeking behavior following a yohimbine challenge [28]. Noteworthy is that it has been also demonstrated that chronic administration of mifepristone in alcohol vapor-exposed rats prevented the escalation of alcohol intake [13]. Consistently, acute mifepristone administration selectively reduced alcohol intake in alcohol-dependent but not in nondependent rats [27]. Moreover, both mifepristone and CORT113176 selectively reduced binge-like ethanol intake in mice selectively bred for high ethanol concentration using drinking in the dark procedures [35]. Finally, it was shown that in nondependent Wistar rats, GR antagonism was more efficacious in female than in male rats [36]. Our results are consistent with these earlier works and confirmed that GR antagonists also reduced alcohol intake in nondependent animals, an effect more robust in female versus male rats [35,36].

Msp rats have long been proposed as an innate phenocopy of a subpopulation of patients that drink excessive amounts of alcohol for tension relief and self-medicating purposes [14]. Earlier studies have demonstrated that this rat line is characterized by two single-nucleotide polymorphisms at the CRF1-R receptor locus, leading to an enhanced expression of CRF1R in different brain regions [16]. Because of this overexpression, they are highly sensitive to stress and show anxious and depressive-like symptoms that are relieved by alcohol consumption [14,16,17]. Recent findings have proved that negative feedback processes regulating HPA responsiveness are impaired in msP versus Wistar rats. Notably, male msP rats showed an innate increase in phosphorylation at the serine site 232 in the CeA, a marker of GR nuclear localization and transactivation [26]. Considering these constitutive alterations in their stress system, and the role of GR in the transition to alcohol dependence, we initially hypothesized that administration of GR antagonists would attenuate alcohol SA more efficaciously in msP rats versus Wistar controls.

In fact, msP rats have long been proposed as a phenocopy of postdependent animals, since they display comorbid symptoms of alcohol preference, high anxiety-like traits and hypersensitivity to stress. Consequently, we proposed that GR antagonism would attenuate the negative affect state that may drive their high alcohol consumption. However, contrary to our expectations, GR antagonists appeared more efficacious in Wistars than in msP rats. Furthermore, we recently reported the GR antagonism also does not alter the innate anxiety-like behaviors in msP rats [30].

There are few possibilities to explain the limited efficacy of GR antagonists in msPs. For instance, in an earlier study we found that male msPs had higher adrenocorticotropic hormone levels but lower circulating CORT, whereas in females, msP rats displayed larger elevation of CORT levels in response to restraint stress versus Wistars. In line with this suggestion, in response to a dexamethasone challenge, msP rats showed a lower reduction in CORT compared to Wistar controls [26]. These findings suggest that msP rats have a different regulation of the HPA axis, and the negative feedback processes modulating its responsiveness are diminished in this rat line. Hence it is possible that an acute injection of GR antagonist is not sufficient to normalize the hormonal equilibrium and to prevent the high alcohol drinking of msP rats. Future studies are needed to evaluate the effects of GR antagonists following chronic administration. A second possibility is that the higher innate GR phosphorylation observed in msP rats may lead to a differential regulation of the intracellular signaling pathways associated with the GR, an effect that may impair binding activity following mifepristone and CORT113176 administration. Thus, it is important to evaluate if transcriptional changes associated with GR activation are different in msPs versus Wistars.

In this study, we also measured plasma CORT levels prior to and after alcohol self-administration. Consistent with the results of earlier work, we found higher basal CORT levels in female compared to male rats [26,37,38]. The highest concentration was detected in female Wistars followed by female msPs. Moreover, we observed that alcohol SA markedly reduced CORT levels in females of both strains, whereas no changes were observed in males. These data are consistent with earlier studies showing that females displayed enhanced glucocorticoids secretion both at baseline and following stress, and after an alcohol challenge [37,38,39,40]. The motivational factors contributing to drinking in males and females may be different, and whether circulating corticosteroid levels may contribute to these discrepancies is unclear. However, it is worth noting that our results indicate that the higher the basal circulating CORT levels, the stronger the inhibitory effect of GR antagonists on alcohol drinking.

Since stress enhances the motivation for alcohol, particularly in female rats, we speculate that their drinking is reduced by GR antagonists via processes that suppress HPA axis function and possibly reduce negative mood associated with steroid hormones dysregulation [41,42].

In summary, our results showed that GR antagonism attenuates alcohol SA, particularly in female rats. Moreover, despite the observation that msPs are more vulnerable to stress and are highly motivated to drink alcohol for tension relieving purposes, they showed a poorer response to GR antagonists.

## 4. Materials and Methods

### 4.1. Animals

Male (N = 25–26/line) and female (N = 28/line) msP and Wistar rats, bred at the animal facility of the University of Camerino, Italy, weighed 250–300 g (male) and 160–200 g (female) at the beginning of the experiments. Rats were housed three per cage in a temperature (20–22 °C) and humidity (45–50%) controlled room with a reverse 12 h light/dark cycle (lights off at 8 AM). During the entire residence in the facility, animals were offered free access to tap water and food pellets (4RF18, Mucedola, Settimo Milanese, Italy). Before the beginning of training, for three days rats were handled 5 min daily by the same operators who performed the experiments. Experiments were conducted during the dark phase of the light/dark cycle. All the procedures were conducted in adherence with the European Community Council Directive for Care and Use of Laboratory Animals and the National Institutes of Health Guide for the Care and Use of Laboratory Animals. Italian Ministry of Health approval 1D580.24.

### 4.2. Drugs

The alcohol drinking solution 10% (*v*/*v*) was prepared by diluting 95% alcohol (F.L.Carsetti, Camerino, Italy) with tap water. Saccharin (Sigma-Aldrich, Milan, Italy) was diluted to 0.2% (*w*/*v*) with tap water. The glucocorticoid and progesterone receptors antagonist mifepristone (Cayman Chemical, Ann Arbor, MI, USA) was dissolved in propylene glycol (Sigma-Aldrich, Milan, Italy). Mifepristone was administered intraperitoneally (i.p.) at the doses of 0.0, 10, 30 and 60 mg/kg in a volume of 1 mL/kg, 90 min before tests. The selective glucocorticoid receptor antagonist CORT113176 (Corcept Therapeutics Incorporated, Menlo Park, CA, USA) was suspended in a vehicle containing 10% dimethylformamide (Sigma-Aldrich, Milano, Italy), 10% Cremophor EL (Sigma-Aldrich, Milano, Italy) and 80% saline. The drug was administered at the doses of 0.0, 10, 30 and 60 mg/kg (i.p). in a volume of 3 mL/kg, 90 min prior the test session. Drug doses were chosen based on published data [24,30].

### 4.3. Self-Administration Apparatus

Self-administration (SA) sessions were conducted in standard operant conditioning chambers (Med Associates, St Albans, VT, USA) enclosed in ventilated sound-attenuating cubicles. Each chamber was equipped with two retractable levers located in the front panel of the chamber with a drinking reservoir placed in between and connected with a syringe pump. A house-light was located on the wall opposite to the levers. Behavioral sessions were controlled and recorded by a windows compatible PC equipped with Med-PC-5 software (Med Associates).

### 4.4. Self-Administration Training

Animals were trained to self-administer 10% (*v*/*v*) alcohol or saccharin 0.2% (*w*/*v*) for five days a week, in 30 min daily sessions under a fixed-ratio 1 (FR1) schedule of reinforcement. Operant sessions started with lever insertion and ended with lever retraction. Responses at the right (active) lever were reinforced with 0.1 mL of fluid (alcohol or saccharin solution) delivered in the drinking reservoir. Rats were trained to alcohol SA using a saccharin-fading procedure [43]. Briefly, during the first five days of training, active lever responses were reinforced with 0.2% (*w*/*v*) saccharin. Next, 8% (*v*/*v*) alcohol was added to saccharin to familiarize rats with alcohol and then alcohol concentration was stepwise increased to 10% (*v*/*v*) and saccharin removed. Starting with alcohol 10% (*v*/*v*) SA, reinforcement delivery was followed by a 5 s time-out (TO), during which the house light was contingently illuminated. During the TO, active lever responses were recorded but not reinforced. Throughout the sessions, responses at the left (inactive) lever were recorded but had no scheduled consequences.

Drug treatments began once a stable SA baseline was established. Approximately three weeks (five SA sessions per week) were necessary to reach a stable baseline of responding.

### 4.5. Experiment 1.1: Effect of Mifepristone on Alcohol Self-Administration in Male and Female msP and Wistar Rats

On test days, male and female msP (N = 10/sex) and Wistar (N = 9–10/sex) rats were injected with mifepristone (10, 30 and 60 mg/kg, i.p.) or its vehicle 90 min before the SA session in a within-subject counterbalanced design. Tests were conducted every fourth day until each rat had received all doses of mifepristone. During the first of the three intervening days, rats remained in their home cage, whereas during the second and third days they performed baseline alcohol SA sessions.

### 4.6. Experiment 1.2: Effect of Mifepristone on Saccharin Self-Administration in Male and Female msP and Wistar Rats

This experiment was conducted on male and female msP (N = 6–8/sex) and Wistar (N = 7–8/sex) rats. The procedure was identical to experiment 1.1 except that the SA fluid was saccharin 0.2% (*w*/*v*).

### 4.7. Experiment 2.1 Effect of CORT113176 on Alcohol Self-Administration in Male and Female msP and Wistar Rats

This experiment was conducted on male and female msP (N = 9–10/sex) and Wistar (N = 10/sex) rats. The procedure was identical to experiment 1.1 except that the selective GR antagonist CORT113176 (0.0, 10, 30 and 60 mg/kg) was used.

### 4.8. Experiment 2.2: Effect of CORT113176 on Saccharin Self-Administration in Male and Female msP and Wistar Rats

This experiment was conducted on male and female msP (N = 9–10/sex) and Wistar (N = 9–10/sex) rats. The procedure was identical to experiment 2.1 except that the SA fluid was saccharin 0.2% (*w*/*v*).

### 4.9. Experiment 3: Blood Corticosterone Levels Following Alcohol Self-Administration in Male and Female msP and Wistar Rats

The effect of alcohol SA on blood corticosterone levels in male and female msP (N = 6/sex) and Wistar (N = 7–8/sex) rats was evaluated. Rats were trained to self-administer alcohol as described above. When a stable alcohol SA baseline was established, blood for corticosterone analysis was collected under a basal alcohol-free condition and immediately after the alcohol self-administration session. The experiment was conducted in a within-subject design and animals were subjected to two blood samplings, one under the basal condition and the other immediately after the self-administration session. At least three days passed between the two blood samplings and sampling order was counterbalanced. Blood was collected by tail nicking. The hypothalamic stress response induced by this sampling procedure is detectable after 3 min [44]; to avoid this confounding factor, we completed sampling within 2 min. Blood was sampled in lithium-heparinized tubes (Sars EDT, Nümbrecht, Germany). Samples were centrifuged at 1500× rcf for 10 min at 4 °C and plasma was collected, aliquoted and stored at −20 °C until further use. Plasma corticosterone levels were determined using enzyme-linked immunosorbent assay (ELISA) (RE52211, IBL International GmbH, Hamburg, Germany) following manufacturer instructions.

### 4.10. Statistical Analysis

All behavioral experiments were analyzed by three-way analysis of variance (ANOVA) with treatment as a repeated measure, and strain and sex as between-subject factors. Active and inactive lever responses were analyzed separately. Behavioral performances of each independent strain/sex group were further analyzed by one-way with factor ANOVAs with treatment as a repeated measure. ANOVAs were followed by Dunnet’s post hoc analysis when appropriate. Significance was conventionally set at *p* < 0.05.

CORT ELISA standards were used to generate an optimalfit 4-parameter standard curve from which sample values were extrapolated. CORT data were analyzed via three-way ANOVA with condition (basal vs. alcohol condition) as the within-subject factor and strain and sex as between-subject factors. Significant effects were explored with Newman-Keuls multiple comparison test. Significance was conventionally set at *p* < 0.05.

All statistical analyses were performed using GraphPad Prism v8.

## Data Availability

The data presented in this study are available upon request to the corresponding author.
